# Alfalfa Responses to Gypsum Application Measured Using Undisturbed Soil Columns

**DOI:** 10.3390/plants6030029

**Published:** 2017-07-11

**Authors:** Rebecca Tirado-Corbalá, Brian K. Slater, Warren A. Dick, Dave Barker

**Affiliations:** 1 School of Environment and Natural Resources, The Ohio State University, Columbus, OH 43210, USA; slater.39@osu.edu; 2 Agro-Environmental Science Department, University of Puerto Rico, Mayagüez, Puerto Rico, 00681, USA; 3 School of Environment and Natural Resources, The Ohio State University, 1680 Madison Avenue, Wooster, OH 44691, USA; dick.5@osu.edu; 4 Department of Horticulture and Crop Science, The Ohio State University, Columbus, OH 43210, USA; barker.169@osu.edu

**Keywords:** alfalfa, gypsum, no-tillage, nutrient uptake, undisturbed soil columns

## Abstract

Gypsum is an excellent source of Ca and S, both of which are required for crop growth. Large amounts of by-product gypsum [Flue gas desulfurization gypsum-(FGDG)] are produced from coal combustion in the United States, but only 4% is used for agricultural purposes. The objective of this study was to evaluate the effects of (1) untreated, (2) short-term (4-year annual applications of gypsum totaling 6720 kg ha^−1^), and (3) long-term (12-year annual applications of gypsum totaling 20,200 kg ha^−1^) on alfalfa (*Medicago sativa* L.) growth and nutrient uptake, and gypsum movement through soil. The study was conducted in a greenhouse using undisturbed soil columns of two non-sodic soils (Celina silt loam and Brookston loam). Aboveground growth of alfalfa was not affected by gypsum treatments when compared with untreated (*p* > 0.05). Total root biomass (0–75 cm) for both soils series was significantly increased by gypsum application (*p* = 0.04), however, increased root growth was restricted to 0–10 cm depth. Soil and plant analyses indicated no unfavorable environmental impact from of the 4-year and 12-year annual application of FGDG. We concluded that under sufficient water supply, by-product gypsum is a viable source of Ca and S for land application that might benefit alfalfa root growth, but has less effect on aboveground alfalfa biomass production. Undisturbed soil columns were a useful adaptation of the lysimeter method that allowed detailed measurements of alfalfa nutrient uptake, root biomass, and yield and nutrient movement in soil.

## 1. Introduction

Gypsum is a common mineral in sedimentary environments [1], and has been used as an amendment on agricultural soils for over 250 years [2,3]. Synthetic gypsum, or flue gas desulfurization gypsum (FGDG), is produced by coal-fired power plants when SO_2_ is scrubbed from the exhaust gases. The production of gypsum in the US was stimulated as a result of the Clean Air Act Amendments of 1990, which mandated a reduction of 6.4 million tons of SO_2_ emissions by electricity utilities from 1990 to 2010. In the USA, more than 30 million Mg of FGDG was generated in 2014 [4]. Of this total, around 49% of FGDG produced was beneficially used in industrial applications, such as highway repairs [5], manufacturing of wallboard, and as a filler ingredient in some food products [6], a small fraction (4%, 1.2 million Mg) was used in agricultural applications, and the remainder FGDG was discarded as waste.

Gypsum has been recognized for its potential to improve soil quality and agricultural productivity [7,8,9]. Gypsum amendments benefit soils (especially sodic or heavy clay soils with poor structure) by promoting soil flocculation, reducing runoff by increasing water infiltration rates, and reducing surface sealing and crusting [10,11,12]. Gypsum applied to the soil surface increases the electrolyte concentration of the infiltrating water, compressing the electric double layer, and providing Ca to the exchange complex where it has selectivity over Mg and Na in most soils [13,14]. Gypsum can also help ameliorate problems associated with subsoil acidity due to its moderate solubility and its removal of toxic Al^3+^ ions by complexation instead of pH adjustment [15,16,17]. Consequently, gypsum treatments enhance deep rooting and improve the ability of plants to access adequate supplies of water and nutrients during drought [18].

Gypsum is an excellent source of Ca and S, two essential macro-elements needed for plant nutrition [7,19]. Many gypsum products, also including FGDG, also provide other essential trace nutrients such as B, Fe, Mo, and Zn. Although FGDG gypsum contains heavy metals such as Pb and Cr [19,20], the concentrations and availability of these metals are generally much lower than regulatory levels specified for soils [21].

Previous studies considered the hydrology and downward transport of gypsum components, and the potential short-term environmental impacts of trace elements in FGDG products, when the products are surface applied or mixed into the top 20 cm of soil [20,22]. However, less information is available about the long-term effects of gypsum, including FGDG, in the whole soil profile [23].

This greenhouse study aimed to provide information of the soil properties and alfalfa growth responses in undisturbed soil columns taken from fields where annual gypsum applications from 0 to 12 years had been applied to two fields managed with no-tillage. The specific objectives were to measure, (1) the movement of gypsum components in soil and (2) the mineral uptake and growth of shoots and roots of alfalfa planted in undisturbed soil columns.

## 2. Results and Discussion

### 2.1. Soil Chemical Properties

**Figure 1:**
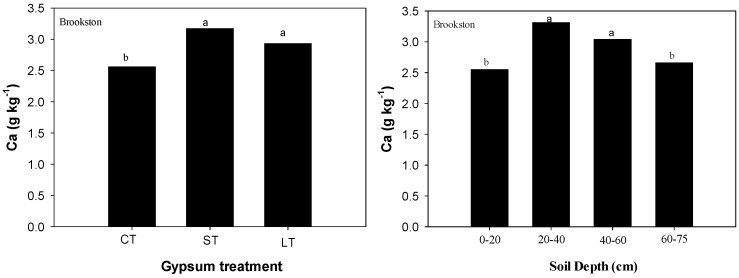
Calcium means values of Brookston soils in for each gypsum treatment (**left**) and in each soil layer (**right**). Means followed by the same letter or no letters for each soil and soil layer are not significantly different by Tukey’s test at *p* < 0.05.

**Figure 2:**
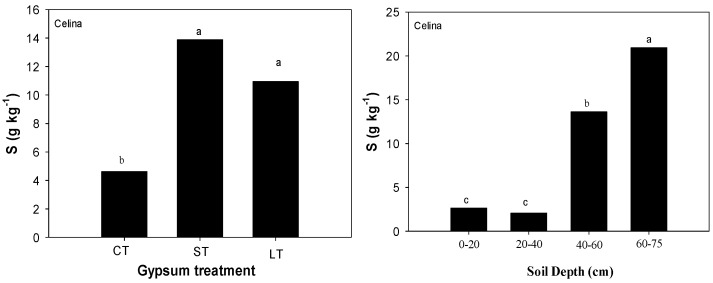
Sulphur mean values of Celina soils for each gypsum treatment (**left**) and in each soil layer (**right**). Means followed by the same letter or no letters for each soil and soil layer are not significantly different by Tukey’s test at *p* < 0.05.

Exchangeable Ca, total S and SO_4_-S were measured but only exchangeable Ca and total S were significantly increased by gypsum application at all soil depths (Table 1). Measurements of exchangeable Ca and total S revealed that dissolution products of surface-applied gypsum had moved throughout the Brookston and Celina profiles compared with CT soils (Table 1 and Table 2). Higher exchangeable Ca and total S were found in gypsum-treated soils compared with CT in both soils (Table 2). For exchangeable Ca, the interaction of gypsum treatment by depth was statistically significant (*p* = 0.035) in Celina soil. Gypsum-treated soils had more exchangeable Ca than CT soils at depths greater than 20 cm. Gypsum-treated soils had at least 0.4 to 1.4 times more exchangeable Ca [LT (3.21 g kg^−1^) and ST- gypsum (3.19 g kg^−1^) treated soils, respectively] at 60–75 cm than CT soils (1.69 g kg^−1^) (Table 2). In Brookston soils, exchangeable Ca main effects (gypsum treatment, soil depth) were statistically significant (Table 1). Higher concentrations of exchangeable Ca were observed in ST and LT gypsum treated soils compared with CT (Figure 1). Also, higher concentrations of exchangeable Ca were observed at 20–60 cm soil depth (Figure 1). For Total-S, the interaction of gypsum treatment by depth was statistically significant (*p* < 0.0001) in Brookston soil (Table 1). Total-S values oscillated between 2.54 and 5.25 g S kg^−1^ in gypsum-treated soils where higher values were encountered in the first 40 cm of soil in ST-gypsum treated soils compared with CT (Table 2). In Celina soils, total- S main effects (gypsum treatment, soil depth) were statistically significant (Table 1). Higher total-S concentrations (>13.6 g·S·kg^−1^) were encountered at deeper soil depths (> 40 cm) (Figure 2). Total-S concentrations were at least two times higher on gypsum-treated soils (13.9 and 10.9 g S kg^−1^ in ST and LT-gypsum, respectively) compared with CT (4.62 g S kg^−1^) (Figure 2).

**Figure 3:**
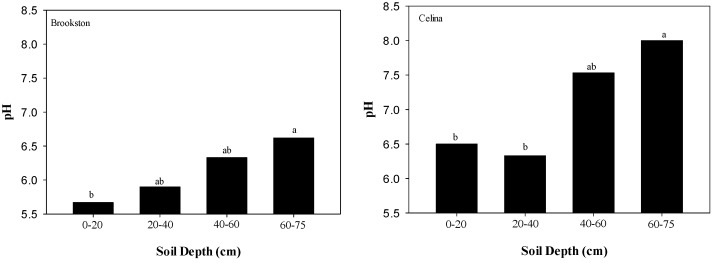
pH values of Brookston and Celina soils in each soil layer. Means followed by the same letter or no letters for each soil and soil layer are not significantly different by Tukey’s test at *p* < 0.05.

**Figure 4:**
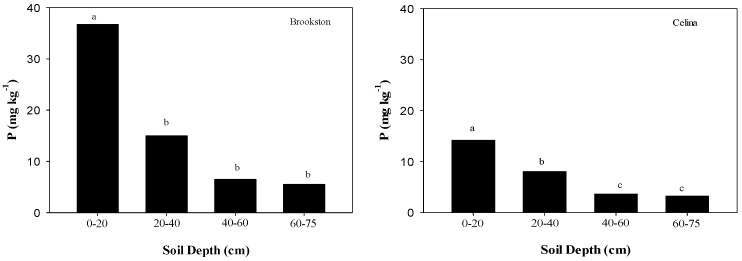
Phosphorous (P) concentration of Brookston and Celina soils in each soil layer. Means followed by the same letter or no letters for each soil and soil layer are not significantly different by Tukey’s test at *p* < 0.05.

No differences in soil pH were observed among gypsum treatments for each soil depth (Table 1); although pH increased with depth in both soils (*p* = 0.03) (Table 1, Figure 3). Also, long-term surface applications of gypsum to Brookston (0.36 dS m^−1^) and Celina (0.39 dS m^−1^) soils did not result in significantly different EC values compared to CT soils (0.54 and 0.46 dS m^−1^ for Brookston and Celina, respectively) (Table 1). Studies by Farina and Channon [24], Toma et al. [17], and Caires et al. [25] found minimal or no effect on soil pH when gypsum was applied to soil systems dominated by permanent charges. Bray-1 P was statistically different for soil depth in both soils (*p* < 0.001) (Table 1). The highest Bray-1 P was found at the 0–20 cm depth of both soils (Figure 4). However, values decreased with depth in both soils (Figure 4).

For total carbon, the interaction of gypsum treatment by depth was statistically significant (*p* < 0.0001) in both soils (Table 1). In the Brookston soil, higher TC values were found for the ST-gypsum treatment in the 0–20 and 20–40 cm depths (TC >20.0 g kg^−1^) but values decreased dramatically below 40 cm (TC <6.7 g·kg^−1^) when compared with the other two treatments (13 < TC > 7 g·kg^−1^) (Table 2). No consistent response of TC was observed in the surface soil layer for the gypsum-treated Celina soil (Table 2). However, ST and LT-gypsum treated Celina soils had higher TC (4.60 and 4.78%, respectively) for the deeper horizons (60–75 cm) when compared with CT soils at the same depths (3.06%) due to presence of calcium carbonates. Soil under the LT-gypsum treatment had higher calcite (calcium carbonates) content as a percentage (%) of total C (8.1 ± 1.1) than the soil under the ST-gypsum treatment (3.9 ± 0.3) and CT soil (1.3 ± 0.2) [23]. Wang and Anderson [26] found applying water with high electrolyte concentration due to presence of gypsum to a calcareous system could increase the calcium concentration in solution above calcite equilibrium, dissolve Ca, Mg and carbonates under saturated conditions, and precipitate some of the dissolution products as secondary carbonates under unsaturated conditions.

Lower concentrations of exchangeable K were observed at depths below 40 cm in both gypsum treatments in the Celina soil compared with the CT treatment (Table 2). No statistical difference was observed for exchangeable K in Brookston soil (Table 1). For exchangeable Mg, the interaction of gypsum treatment by depth was statistically significant in both soils (Table 1 and Table 2). Lower concentrations of exchangeable Mg were observed in LT-gypsum treated Brookston soil compared with CT (Table 2). Although, in Celina soils, lower concentrations of exchangeable Mg were observed at depths below 40 cm in both gypsum treatments in the Celina soil compared with the CT treatment (Table 2). Lower concentrations of exchangeable K and Mg are likely due to Mg and/or K replacement on the exchange sites by Ca. It resulted in reductions in exchangeable Mg and K, mainly in the upper part of the profile [9,24,27].

### 2.2. Plant and Root Responses

The average cumulative dry weight and average plant height for the Brookston soil were 2561 g·m^−2^ and 17.2 cm, respectively; and for Celina soil were 1,820 g m^−2^ and 14.4 cm, respectively. However, no significant difference among treatments (*p* > 0.28) was observed for both soils. A similar result for yield was found by Sloan et al. [20] where FGDG applied to alfalfa in the upper Midwest (i.e., Minnesota, Wisconsin, Michigan, North and South Dakota, Indiana, Illinois, Iowa, and northern Ohio) at agronomic rates (up to 3,750 kg ha^−1^) did not affect yield, but increased the S content of alfalfa plants relative to alfalfa grown on untreated soil. In addition, O’Leary and Rehm [28] found alfalfa dry weight was not affected by gypsum application on silt loam soils in Wisconsin. The higher Brookston plant yield and height were attributed to the Brookston soil having a more optimum amount of K for plant growth. The optimum soil K for most crops in Ohio is 100 to 200 mg·K·kg^−1^ soil [29], and alfalfa has a particularly high K requirement [22]. We found that in the Brookston soil-K concentration was higher than 100 mg·kg^−1^ in all treatments in the first 40 cm of soil (Table 2). However, for the Celina soil, the CT and LT gypsum treatments resulted in soil K concentrations that were lower than the optimum in the 0–20 cm soil depth.

Total root biomass for alfalfa in each soil was significantly improved by gypsum application (Table 1 and Table 3). However, the positive gypsum effect on root biomass was mostly restricted in the 0–10 cm soil depth where approximately 60% of the root biomass occurred (Table 3). At deeper depths, there was no significant effect of gypsum. In our experiment, water was not limited. Thus, root exploration into deeper soil layers was not stimulated by drought conditions (Table 3). The increased root biomass in the 0–10 cm depth would likely have made uptake of nutrients from this nutrient rich soil layer more efficient. If gypsum had stimulated root growth deeper into the soil profile during drought conditions, as reported by Wendell and Ritchey [30], then the combination of improved nutrient and water uptake brought about by improved rooting depth with gypsum additions may lead to crop yield increases [31].

### 2.3. Alfalfa Nutrient and Trace Element Concentrations

#### 2.3.1. Major Nutrients

Calcium is an important nutrient for good root growth [17,32], especially responsible for strengthening cell walls and for developing root tips (Fisher, 2011). Calcium concentrations in gypsum-treated soils (Table 2) were higher compared with CT soils in both soil series but alfalfa tissue Ca concentrations were not statistically different (*p* > 0.05), even though large amounts of Ca were added to the soil as gypsum (Table 4). A similar result for Ca concentration in alfalfa was found by Chen et al. [22] when Wooster silt loam (Typic Fragiudalf) soil was treated with FGDG, ag-lime, or left untreated. The authors attributed these opposite-to-expected results to the Ca uptake rate which did not surpass the rate of plant growth and accumulation of this element in the plant tissue.

Higher tissue concentrations of S relative to the CT were measured in alfalfa receiving ST and LT gypsum treatments in the alfalfa growing in the Brookston soil, but not the Celina soil (Table 4). This result was not surprising as gypsum is an excellent source of S and its application to soil is expected to be reflected in plant tissue, especially for crops growing in S-deficient soils. However, the addition of S had no positive or negative effect on cumulative dry weight (Table 3).

Significantly higher K concentration was measured in alfalfa tissue for the ST treatment, compared to the control, especially in Celina soil (Table 4). In the LT treatment, the tissue concentration of K in alfalfa was maintained in the Celina soil (Table 4), even though the exchangeable K concentrations in the soil were decreased by gypsum and soil depth (Table 2). The gypsum seemed to have displaced the K and made it more available to alfalfa. For Mg, lower tissue concentrations were generally observed in alfalfa growing in both the gypsum-treated Brookston and Celina soils, compared to the CT (Table 4). Similar results were found by Chen et al. [22] where the Mg concentration in alfalfa tissue significantly decreased when soil was treated with FGDG containing vermiculite and slightly decreased when treated with FGDG by-products containing perlite compared to control. The rate of Mg uptake tends to be depressed by its competition with cations such as Ca, K, and Mn [22,33].

No statistical difference (*p* > 0.05) was found among treatments in both soils for alfalfa tissue N (Table 4). A lower tissue P concentration was found for the LT treatment (2.94 g·kg^−1^) compared with the ST treatment (3.62 g·kg^−1^) in the Brookston soil. No statistical difference was found among treatments in the Celina soil. Alfalfa plants growing in the Celina soils were slightly P deficient (<2.6 g·P·kg^−1^) based on the critical tissue ranges given by Pickerton et al. [34]. The possible P deficiency is important to note because there has been concern expressed that gypsum may reduce available P for plant uptake, but this seems not to be the case for Celina soils. Brauer et al. [35] found that gypsum did not change soil test *P* values.

#### 2.3.2. Selected Elements

The FGDG provided B (~26.7 mg·kg^−1^), an essential element for plant growth, so that higher B concentrations were found in alfalfa receiving ST and LT gypsum treatments in each soil (Table 5). Alfalfa growing in gypsum-treated soil had greater tissue B concentrations than alfalfa growing in soil that received no gypsum (Table 5); however, these concentrations were not considered phytotoxic [36]. Jones et al. [37] reported that alfalfa leaf concentrations above 30 mg·kg^−1^ were sufficient for alfalfa.

Concentrations of Cu, Mn, Mo, and Zn in alfalfa tissue (Table 5) were affected by the gypsum treatments and within accepted concentrations for healthy plants in Brookston soil [33]. In the case of Cu and Mn, the concentrations were lower under LT and ST application of gypsum compared to CT treatment (Table 5). Higher concentrations of Mo and Zn were found in alfalfa grown in the Brookston soil that received the ST gypsum treatment. Although there were no statistically significant treatment-related concentration differences for Cu, Mn, and Ni in alfalfa growing in the Celina soils (Table 5).

Alfalfa tissue Mo concentration was sufficient for plant growth (Table 5). Molybdenum concentrations are particularly important to legumes because Mo is a constituent of the nitrogenase enzyme. Adequate levels of Mo in aboveground alfalfa tissue have been reported to range from 0.15 to 1.30 mg·kg^−1^ [34].

The Cu concentrations in alfalfa tissues were mostly below the critical deficiency levels (Table 5). The critical deficiency level of Cu in vegetative plant parts generally ranges from 1–5 mg·kg^−1^ dry weight, depending on plant species, plant organ, developmental stage and N supply [38]. Based on Pickerton et al. [34], our plants might have experienced Cu deficiency (<4 mg·kg^−1^). In general, the critical deficiency level in the youngest emerged leaf is less affected by environmental factors than older leaves.

There were no significant differences among gypsum treatments for Al and Cr concentrations in alfalfa (Table 5). Higher concentrations of Cd were found under ST-gypsum treated soils in Brookston soil, while lower concentrations of Ba were found in alfalfa growing in the Brookston soil receiving LT–gypsum (Table 5). There was no statistical difference among treatments for tissue Ba concentration for alfalfa growing in the Celina soil (Table 5). Concentrations of As (1.28 mg·kg^−1^), Pb (0.77 mg·kg^−1^), and Se (2.32 mg·kg^−1^) were below detection limits which are shown here in parentheses. Chen et al. [7] found similar results to our outcomes for concentrations of Cr, Pb, and Se which exhibited no effect of FGDG by-products or ag-lime treatments. However, plant Al, Ba and Cd concentrations significantly decreased in alfalfa growing in plots treated with FGDG by-products or ag-lime.

## 3. Materials and Methods 

### 3.1. Study Site

In late March to the beginning of April 2008, twenty-four, 30.5 cm i.d. and 75 cm long undisturbed soil columns [schedule 40 polyvinyl chloride (PVC) pipes] were collected in a commercial farm from two nearby soil series with three gypsum management systems (two soil series × three gypsum treatments = six fields) in a uniform prime agricultural landscape in southwest Ohio, USA (39°45’17” N, 84°40’28” W). The soil series were Brookston (fine-loamy, mixed, superactive, mesic Typic Argiaquolls, 1% slope) and Celina (fine, mixed, active, mesic Aquic Hapludalfs, <6% slope) soil series associations that are commonly found in the glaciated Ohio landscape and southeastern Indiana. Twelve columns of each soil series were collected. Fields from both soil series had been managed by the farmer in a similar cropping history since 1996, comprising rotations of corn (*Zea mays* L.) and soybean (*Glycine max* L.). Only corn received annual fertilizer application (178, 43, 65, and 11 kg·ha^−1^ of N (Ammonium nitrate, 34-0-0), P (Monoammonium Phosphate, 11-52-0), K (Potassium oxide, 0-0-60), and S (Ammonium thiosulfate, 12-0-0-26), respectively, during each April since 1996). The farm had been converted from conventional (i.e., chisel tillage) to no-tillage cultivation in 1996.

The three gypsum treatments were: (1) control treatment (CT), no gypsum application; (2) short-term treatment (ST), consisting of annual applications of gypsum at a rate of 1680 kg·ha^−1^ over the previous 4 years for a total of 6720 kg·ha^−1^ (i.e., since 2004); and (3) long-term treatment (LT), consisting of annual applications of gypsum at a rate of 1680 kg·ha^−1^ for the previous 12 years for a total of 20,200 kg·ha^−1^. For the LT treatment, the first six gypsum applications were waste drywall gypsum (WDG). The subsequent six applications of gypsum on the LT fields and all four applications on the ST fields were made using FGDG. All gypsum applications for the ST and LT treatments were applied each year during the month of December. Both WDG and FGDG were applied to the fields using a double spinner lime spreader.

The FGDG was of high purity with <3% water insoluble residues and trace element concentrations. The chemical contents of FGDG determined by Midwest Laboratories from samples collected from Zimmer Station Wet FGDG By-Products (Station from where the farmer gets the FGDG) were 19.8, 0.02 and 16.2% for Ca, Mg and S, respectively; and the concentrations of P and B were 16.7 and 26.7 mg·kg^−1^, respectively. Dontsova et al. [19] reported that WDG concentrations were 21.9, 0.22 and 18.9% for Ca, Mg and S, respectively. The waste drywall gypsum P and B concentrations were 51.6 and 7.3 mg·kg^−1^, respectively. Also, Don Dontsova et al. [19] reported that FGDG had a smaller and more uniform particle size (40 µm), a lower cost, and better flow characteristics than the WDG.

### 3.2. Field Sampling Prior Soil Columns Collection

Composite duplicate soil samples from each field were collected from four depths (0–20, 20–40, 40–60, and 60–75 cm) using a 3” regular auger, air-dried and sieved by hand through a 2-mm screen prior to the collection of soil columns to characterize the soil. Exchangeable Ca^2+^, Mg^2+^, and K^+^ were extracted using 1 mol·L^−1^ NH_4_OAc [39] and available P by Bray-1 extractant [40]. Soil pH was measured in a 1:1 (*v*:*v*) soil–water mixture [41]. Total S was determined via inductively coupled plasma spectrometry (ICP) (Teledyne Leeman Labs Prodigy Dual, Hudson, NH) after Perchloric acid digestion [42,43]. Soluble Sulfate-S was measured by ion chromatography (Dionex DX 120, Sunnyvale, CA, USA) following monocalcium phosphate extraction [44]. Soil electrical conductivity (EC) was determined in a 1:5 (*v*:*v*) soil–water suspension [45]. Triplicate soil samples from each treated field area and soil depth were used to measure total C (TC) via the combustion method [46]. Calcium carbonate equivalence (CCE) were measured by the gasometric method of Dreimanis [47] employing a Chittick apparatus.

### 3.3. Soil Column Collection

Four columns from each of the three gypsum treatments for two soil series (a total of 24 columns) were collected from the commercial farm using a free standing, portable hydraulic ram designed by Hutton et al. [48]. The hydraulic ram was powered using a tractor mounted 775–900 kg skid loader (model 317, John Deere Manufacturing Company, Moline, IL, USA) with closed-center configuration as described in Tirado-Corbalá et al. [23]. All samples in each site were collected within a close geographical radius of 0.25 km to reduce possible spatial variability. Once the PVC pipes were inserted, a backhoe was used to dig a trench between two lines of columns and the columns lifted from the soil. Once all the soil columns were lifted, excess soil from each column was removed from the outside of the PVC pipes followed by the installation of an end cap with 5–7 cm layer sand to prevent compaction or any disturbance of the soil during transportation. The secured soil columns were loaded onto a truck and transported to a greenhouse at The Ohio State University, Columbus, OH.

### 3.4. Alfalfa Greenhouse Experiment Using Collected Soil Columns

Although 24 soil columns were moved to the greenhouse, damage to some columns resulted in only 18 columns being used for the soil columns study (July 2008 to January 2009). A block factorial design was used, that included all combinations of three gypsum treatments (CT, ST, and LT), two soils (Celina and Brookston), and three replicates. Treatments were randomized within each replicate block. “Evergreen-3” alfalfa was grown from 200 seed column^−1^ planted on July 1, 2009, that was subsequently thinned to 150 seedlings column^−1^. This was approximately double the rate for a field planting due to the short-term nature of this experiment, in which plants did not attain the full size that might be expected from mature field plants. Alfalfa seed had been pretreated with a commercial preparation of *Sinorhizobium meliloti* to ensure nodulation.

For the first three-month growing period (July–September, 2008), water was applied in the greenhouse at the same rate as the daily average precipitation was calculated using 30 years of data. Climatological data for Eaton, OH, the closest weather station to the fields where the soil columns were collected, were obtained from the National Oceanic and Atmospheric Administration (NOAA). For the subsequent three months (October–December, 2008), water was applied at four times the average 30-year precipitation rate to simulate wetter conditions and greater leaching. A total of 2063 mm of water was applied to each soil column during the six-month period.

Natural illumination was used from July to October 2008 and artificial illumination was used from 14 October 2008 to 20 January 2009 to provide a minimum day length of 14 h. Temperature was recorded during the research period to ensure the greenhouse temperature was 23.0 ± 2.0 °C.

### 3.5. Alfalfa Analysis

Alfalfa yield was measured six times during the study (2008–2009) as total aboveground (5-cm cutting height) dry biomass per column. The first harvest was performed at the first flowering. Five subsequent harvests were made at about 35-d intervals. Alfalfa height was recorded the day before each harvest. Alfalfa tissue from each harvest was dried at 60 °C for 4 d and weighed to determine dry weight harvest^−1^ and total dry weight summed across all six harvests. At 180 days after seeding alfalfa (harvest #6), plant tissue (10 g) from each soil x gypsum treatment combination was grounded to pass through a sieve with 0.1 mm openings and analyzed for P, K, Ca, Mg, S, Fe, Mn, Al, Na, B, Cu, Mo, and Zn by ICP spectrometry (Teledyne Leeman Labs Prodigy Dual view ICP Hudson, NH) after Perchloric acid digestion [43]. Total N and C were determined by combustion analysis.

After the sixth harvest, two of the three columns of each treatment were vertically bisected, using a reciprocating saw, to allow roots to be sampled for measurement of root biomass. Soil from one half of each vertically bisected column was sectioned into five horizontal depth increments (0–10, 10–20, 20–40, 40–60, and 60–75 cm). The roots within each section were gently washed free of soil with tap water and given a final rinse with deionized water. Later, the cleaned roots were dried at 65 °C in a paper bag and weighed to determine the dry mass per depth, and total dry mass of each column/treatment.

### 3.6. Statistical Analysis

For statistical purposes, the gypsum application duration (i.e., 0, 4, or 12 years) was treated as being randomly assigned to field areas and variability was assumed to be primarily due to soil and gypsum rate. For soil chemical properties analysis and root dry weight (per depth), gypsum treatment (0, 4, or 12 years duration) and soil depth increment were treated as fixed factors and replicate was considered as random factor. The analysis of variance (ANOVA) was performed by soil. The alfalfa growth response variables that were statistically analyzed, as affected by treatments in the soil columns, were cumulative plant dry weight, plant height, total root dry weight, and alfalfa shoot nutrient concentrations. The ANOVA and Tukey’s test (*p* < 0.05) for mean comparisons were performed using the Statistical Analysis System JMP Version 9.0 (SAS Institute, Cary, NC, USA).

## 4. Conclusions

In summary, the increased root mass stimulated by the gypsum addition suggests that under water and/or nutrient limiting conditions, the alfalfa would respond favorably to gypsum application. Positive soil fertility and alfalfa growth responses were observed, but the responses were not strong or always consistent across the two soils or under the different total amounts of gypsum application. Application of gypsum had no measurable effect on alfalfa yield and generally little effect on macronutrient status, except for increased S uptake. Also, because gypsum is an excellent source of S, the application of gypsum to soils where availability of this element is limited should lead to improved alfalfa production.

## Figures and Tables

**Table 1:** Probability levels for testing of treatment effects calculated from analysis of variance of alfalfa root dry weight and chemical properties of two Ohio soil series treated with three levels of gypsum.

Soil	pH	P-Bray 1	TC ^†^	EC	Ca	Mg	K	Total-S	SO_4_-S	Root Dry Weight
*Brookston*										
Gypsum	0.629 ^‡^	0.118	0.0003	0.139	0.002	0.0004	0.173	0.0001	0.814	<0.0001
Depth	0.030	<0.001	<0.0001	0.959	0.005	<0.0001	0.141	0.0007	0.698	0.045
Gypsum Depth	0.990	0.333	<0.0001	0.997	0.696	0.028	0.550	<0.0001	0.955	<0.0001
*Celina*										
Gypsum	0.239	0.246	<0.0001	0.528	<0.0001	0.0009	0.060	0.012	0.680	<0.0001
Depth	0.005	<0.0001	<0.0001	0.954	<0.0001	0.004	0.001	0.0001	0.958	<0.0001
Gypsum Depth	0.934	0.112	<0.0001	0.904	0.035	0.002	0.018	0.082	0.979	<0.0001

^†^ TC= Total carbon, EC=Electrical conductivity; ^‡^ All values were compared to an alpha of 0.05.

**Table 2:** Total (C) and macronutrients (Ca, Mg, K and S) in two Ohio soil profiles receiving three levels of gypsum.

Soil/Depth	TC ^†^			Ca			Mg			K			S		
cm	g·kg^−1^			g·kg^−1^			g·kg^−1^			g·kg^−1^			g·kg^−1^		
*Brookston*	CT ^a^	ST	LT	CT	ST	LT	CT	ST	LT	CT	ST	LT	CT	ST	LT
0–20	19.0 aA^‡^	20.5 aA^§^	17.7 aA	2.37	2.66	2.61	4.64 bC	6.05 aC	2.96 cC	1.49	1.53	1.30	4.47 bA	5.25 aA	3.77 cA
20–40	13.6 bB	20.6 aA	14.9 bA	3.03	3.76	3.11	8.31 aA	7.41 bB	5.06cB	1.42	1.12	1.12	2.84 cB	4.60 aB	3.93 bA
40–60	12.0 aB	6.7 bB	13.2 aB	2.56	3.39	3.18	8.31 aA	8.04 bA	7.09 cA	1.23	0.88	1.24	2.07 cC	3.34 aC	3.00 bB
60–75	7.1 bC	3.5 cB	11.8aB	2.28	2.85	2.85	7.65 aB	7.19 bB	7.32 bA	1.24	0.86	1.28	2.10 bC	2.54 aD	2.85 aB
*Celina*															
0–20	12.2 aB	11.3 aC	13.0 aC	1.14 aA	1.76 aC	1.52 aC	2.25 bC	4.92 aA	1.87 bC	0.67 bB	1.22 aA	0.70 bA	2.51	2.73	2.67
20–40	4.4 cC	31.9 aB	6.9 bD	1.20 cA	2.04 aBC	1.59 bC	4.28 aB	5.32 aA	2.51 bB	0.67 bB	0.80 aB	0.65 bA	1.65	2.06	2.47
40–60	3.4 cC	44.1 aA	22.7 bB	1.88 cA	3.07 aAB	2.10 bB	7.78 aA	3.05 bB	3.38 bA	0.87 aA	0.43 cC	0.52 bA	5.44	23.6	11.8
60–75	30.6 bA	46.0 aA	47.8 aA	1.69 bA	3.19 aA	3.21aA	4.19 aB	1.77 bC	1.61 bC	0.52 aC	0.42 bC	0.26 cB	8.86	27.1	26.8

^†^ CT = control, no gypsum application; ST = four years of gypsum application at 1680 kg·ha^−1^·year^−1^, totaling 6720 kg·ha^−1^, and LT = 12 years of gypsum application at 1680 kg·ha^−1^·year^−1^, totaling 20,200 kg·ha^−1^, TC= Total carbon; ^‡^ Means followed by the same lower case letter or no letter in a row between treatments for each chemical variable are not significantly different by Tukey test at *p* < 0.05; ^§^ Means followed by the same upper case letter or no letter in a column for each treatment on each chemical variable are not significantly different by Tukey test at *p* < 0.05.

**Table 3:** Root dry weight per depth and total of alfalfa plants grown in a greenhouse for 180 days in intact cores of two Ohio soils treated with three levels of gypsum.

Soil/Depth	CT ^†^	ST		LT
cm	kg·m^−3^			
*Brookston*				
0–10	83.3 b^‡^A		110 aA^§^	129 aA
10–20	43.3 aB		62.0 aB	59.9 aB
20–40	19.1 aC		14.9 aC	13.2 aC
40–60	12.1 aC		9.9 aC	12.0 aC
60–75	4.7 aC		4.2 aC	4.7 aC
Total ^¶^	162 b		201 a	219 a
*Celina*				
0–10	70.0 bA		76.0 bA	125 aA
10–20	41.0 aB		46.0 aB	55.5 aB
20–40	10.0 aC		9.9 aC	12.0 aC
40–60	12.0 aC		8.6 aC	4.9 aC
60–75	4.5 aC		3.9 aC	4.7 aC
Total	138 b		144 b	202 a

^†^ CT = control, no gypsum application; ST = four years of gypsum application at 1680 kg·ha^−1^·year^−1^, totaling 6720 kg·ha^−1^, and LT = 12 years of gypsum application at 1680 kg·ha^−1^·year^−1^, totaling 20,200 kg·ha^−1^; ^‡^ Means followed by the same lower case letter or no letter in a row between treatments are not significantly different by Tukey test at *p* < 0.05; ^§^ Means followed by the same upper case letter or no letter in a column for each treatment are not significantly different by Tukey test at *p* < 0.05; ^¶^ Total root dry weight for the root samples collected from 0–75 cm soil depth.

**Table 4:** Mean concentrations of major plant nutrients (Ca, K, Mg, P, S, and N) in alfalfa shoots grown in a greenhouse at the end of harvest (180 days after planting) in intact cores of two Ohio soils treated with three levels of gypsum.

Soil	Treatment ^†^	Ca	K	Mg	P	S	N
		g·kg^−1^					%
Brookston	CT	15.3 ^‡^	23.4 ab	3.60 a	2.88 b	4.34 b	4.76
	ST	15.2	27.2 a	3.63 a	3.62 a	4.69 a	4.82
	LT	15.4	22.2 b	3.18 b	2.94 b	4.78 a	4.74
	P > F	0.68	0.035	0.006	0.002	0.005	0.82
Celina	CT	18.0	15.8 b	4.76 a	2.33	4.73	4.68
	ST	16.3	21.9 a	4.12 b	2.49	4.68	4.66
	LT	18.4	18.6 a	3.75 c	2.15	5.04	4.53
	P > F	0.17	0.007	0.005	0.42	0.17	0.08

^†^ CT= Control, no gypsum application, ST = 4 years annual gypsum application (6,720 kg/ha total; 1,680 kg/ha per year) and LT = 12 years annual gypsum application (20,200 kg/ha total; 1,680 kg/ha per year), and N= Total nitrogen; ^‡^ Means followed by the same letter or no letters in a column for each soil are not significantly different by Tukey’s test at *p* < 0.05.

**Table 5:** Mean concentrations of selected elements (Al, B, Ba, Cd, Cr, Cu, Fe, Mn, Mo, Ni, and Zn) in alfalfa shoots grown in a greenhouse at the end of harvest (180 days after planting) in intact cores of two Ohio soils treated with three levels of gypsum.

Soil	Treatment ^†^	Al	B	Ba	Cd	Cr	Cu	Fe	Mn	Mo	Ni	Zn
		mg·kg^−1^										
*Brookston*	CT	298 ^‡^	27.3 b	27.1 b	0.26 b	0.71	1.36 a	341	88.6 a	1.43 b	4.10	341
	ST	649	33.9 a	30.3 a	0.48 a	1.05	1.94 a	445	41.4 b	3.05 a	3.25	445
	LT	437	36.4 a	21.4 c	0.19 b	0.64	0.85 b	451	51.4 b	0.92 b	3.60	451
	P > F	0.49	0.012	0.0003	0.0013	0.55	0.004	0.72	0.003	0.0001	0.093	0.72
												
*Celina*	CT	206	14.3 b	0.90	0.10	0.73	1.55	300	76.2	1.65	2.89	300
	ST	411	19.6 a	0.90	0.14	0.79	0.87	446	77.9	0.73	3.05	446
	LT	226	26.6 a	0.90	0.06	0.65	0.59	367	73.8	1.26	2.31	367
	P > F	0.59	0.018	N/A	0.07	0.89	0.32	0.74	0.79	0.11	0.19	0.74

^†^ CT = Control, no gypsum application, ST = 4 years annual gypsum application [6720 kg/ha total; 1680 kg/ha per year] and LT = 12 years annual gypsum application [20,200 kg/ha total; 1680 kg/ha per year]; ^‡^ Means followed by the same letter or no letters in a column for each soil are not significantly different by Tukey’s test at *p* < 0.05.
